# Integrated analysis identifies oxidative stress genes associated with progression and prognosis in gastric cancer

**DOI:** 10.1038/s41598-021-82976-w

**Published:** 2021-02-08

**Authors:** Zhengyuan Wu, Lin Wang, Zhenpei Wen, Jun Yao

**Affiliations:** 1 Department of Orthopedics Trauma and Hand Surgery, The First Affiliated Hospital of Guangxi Medical University, Nanning, 530021 China; 2 Department of Bone and Joint Surgery, The First Affiliated Hospital of Guangxi Medical University, Nanning, 530021 China; 3 Guangxi Collaborative Innovation Center for Biomedicine, Guangxi Medical University, Nanning, 530021 China

**Keywords:** Cancer, Computational biology and bioinformatics, Genetics

## Abstract

Oxidative stress (OS) reactions are reported to be associated with oncogenesis and tumor progression. However, little is known about the potential diagnostic value of OS in gastric cancer (GC). This study identified hub OS genes associated with the prognosis and progression of GC and illustrated the underlying mechanisms. The transcriptome data and corresponding GC clinical information were collected from The Cancer Genome Atlas (TCGA) database. Aberrantly expressed OS genes between tumors and adjacent normal tissues were screened, and 11 prognosis-associated genes were identified with a series of bioinformatic analyses and used to construct a prognostic model. These genes were validated in the Gene Expression Omnibus (GEO) database. Furthermore, weighted gene co-expression network analysis (WGCNA) was subsequently conducted to identify the most significant hub genes for the prediction of GC progression. Analysis revealed that a good prognostic model was constructed with a better diagnostic accuracy than other clinicopathological characteristics in both TCGA and GEO cohorts. The model was also significantly associated with the overall survival of patients with GC. Meanwhile, a nomogram based on the risk score was established, which displayed a favorable discriminating ability for GC. In the WGCNA analysis, 13 progression-associated hub OS genes were identified that were also significantly associated with the progression of GC. Furthermore, functional and gene ontology (GO) analyses were performed to reveal potential pathways enriched with these genes. These results provide novel insights into the potential applications of OS-associated genes in patients with GC.

## Introduction

Gastric cancer (GC), which was the third leading cause of cancer mortality until 2018, exhibits the fifth largest incidence rate worldwide, and remains a serious threat to human health^1,2^. The occurrence and progression of GC is a complicated multi-step process involving various genetic and epigenetic risk factors^3^, where *Helicobacter pylori* infection is the most common^4^. Owing to the lack of specific symptoms, most patients with GC are diagnosed at an advanced stage, and thus, have a significantly poor 5-year survival rate^5^. Presently, the optimal management of patients with GC is surgical resection, although its overall 5-year survival rate is only 20–25%. Moreover, approximately half of the patients with GC who receive adjuvant therapy experience systemic or local tumor recurrence^6^. Unfortunately, GC diagnosis and treatment currently does not meet the needs for earlier diagnosis and longer survival time. Hence, exploration of novel biomarkers that provide increased predictive value is urgently required to improve prognostication for GC.

Currently, the unequivocal mechanism leading to GC carcinogenesis remains poorly understood; however, it is proposed that oxidative stress is an important factor in driving tumorigenesis and cancer progression through excessive production of reactive oxygen species (ROS)^7–9^. As a characteristic of OS, ROS comprises free radicals or reactive nonradical species, including singlet oxygen, hydrogen peroxide (H2O2), and superoxide anion^10^. Additionally, ROS is dramatically elevated in patients with GC^11^. In the absence of scavenging potential, ROS leads to genotoxicity and induces DNA damage^12,13^. Moreover, accumulated DNA damage eventually induces various genomic mutations and initiates tumorigenesis^14,15^. As a recognized risk factor of GC, increasing evidence indicates a positive association between *H. pylori* infection and gastric adenocarcinoma due to increased OS^16^. *Helicobacter pylori* infection generates ROS by activating various oxidant-producing enzymes, including inducible nitric oxide synthase and nicotinamide adenine dinucleotide phosphate (NADPH) oxidase^17^, and subsequently activates several pathways like Wnt, mTOR, and Ras, to initiate GC^18–20^. These studies have clarified that OS is closely correlated with the progression of GC. Nevertheless, the prognostic value of these OS genes in GC prognosis prediction is largely unclarified, and the underlying mechanisms require further validation.

GC diagnostic methods mainly depend on imaging tests, molecular diagnostics, and histopathological examination. Only a small fraction of OS-related genes has been studied intensively and are known to play a key role in GC progression. Recently, large-scale tumor genome profiles have provided gene expression data, which provides an excellent chance to identify potential molecular markers^21,22^. And bioinformatic analysis of OS genes in this study might also help to discover new GC diagnostic or prognostic markers to screen for innovative treatment targets. Herein, GC RNA-sequencing data and the corresponding clinical information were downloaded from The Cancer Genome Atlas (TCGA) and Gene Expression Omnibus (GEO) databases, and several prognosis-annotated OS genes were selected to construct a risk model. In addition, the relationship between the expression of OS genes and GC progression was examined using the weighted gene co-expression network analysis (WGCNA) method, which is a bioinformatics method that describes the relevance of gene sets and clinical traits between different samples^23,24^. Gene ontology (GO) and Kyoto Encyclopedia of Genes and Genomes (KEGG) pathway enrichment analyses were also performed to probe the underlying mechanisms of OS genes in GC. Ultimately, a cluster of OS genes involved in the prognosis and progression of GC was identified, some of which might be developed as potential prognostic and diagnostic biomarkers in the future.

## Material and methods

### Data acquisition and differentially expressed OS genes (DEOSGs)

The RNA-sequencing dataset comprised 375 GC samples and 32 normal gastric tissues with corresponding clinical information downloaded from TCGA database (https://portal.gdc.cancer.gov/) on May 3, 2020. To identify DEOSGs, 1399 OS protein domains were extracted from GeneCards (https://www.genecards.org) with a relevance score ≥ 7 (Supplement file 1), and further preprocessed with the limma package in view of a false discovery rate (FDR) < 0.05 and |log_2_ fold change (FC)|≥ 1, in accordance with previously reported methods^25^. Meanwhile, genes with an average count value of < 1 were eliminated. This yielded 279 DEOSGs for further analysis. Additionally, gene profiles and clinical information of 433 patients with GC from the GSE84437 dataset (https://www.ncbi.nlm.nih.gov/geo/) were used as a validation cohort.

### GO and KEGG pathway enrichment analysis

GO enrichment and KEGG pathway analysis^26–28^ were applied to systematically investigate the biological functions of selected DEOSGs using the Database for Annotation, Visualization, and Integrated Discovery (DAVID) version 6.8^29^. GO analysis comprehensively comprised three terms: biological process (BP), cellular component (CC), and molecular function (MF). *P* and FDR values < 0.05 were considered significantly different.

### Protein–protein interaction (PPI) network construction and module screening

The PPI information among all DEOSGs was identified using the search tool for the retrieval of interacting genes/proteins (STRING) online platform (http://www.string-db.org/)^30^, and subsequently, their interactions were imported into the Cytoscape 3.7.0 software to construct and visualize a PPI network. The Molecular Complex Detection (MCODE) plug-in was also used to elect the virtual modules and hub genes in the PPI network with both MCODE scores and node counts > 5^31^. *P* < 0.05 was considered the significant threshold.

### Prognostic model construction and efficacy evaluation

All hub DEOSGs in the key modules were subjected to univariate Cox regression analysis using the survival R package to explore the relationship between each gene and patients’ overall survival; the genes with *P* < 0.05 were identified as prognosis related DEOSGs. Subsequently, these candidate genes were integrated into the least absolute shrinkage and selection operator (LASSO) regression^32^ to construct a potential risk signature of patients with GC. The formula for each sample’s risk score was calculated as follows: $\mathrm{risk score}=\mathrm{\Sigma expgenei}*\mathrm{\beta i}$, where expgene represents the relative expression value of OS genes, and β represents the regression coefficient.

Based on the LASSO prognostic model, patients were categorized into high- and low-risk groups, and the Kaplan–Meier method and log-rank test using R Bioconductor survival package were further conducted to compare the overall survival between the two subgroups. Additionally, the survivalROC and timeROC packages in R were calculated to validate the predictive accuracy and ability of the signature^33^, and univariate and multivariate Cox regression analyses were also performed to evaluate the relationship between clinical characteristics and risk score. Finally, a nomogram incorporating calibration plots was constructed to forecast the clinical outcome of patients with GC using the RMS R package^34^. All methods were also included in the GEO cohort to confirm the prognostic performance of this model.

### Hub gene evaluation

To clarify the differential expression of 11 hub DEOSGs at a translational level, the Human Protein Atlas (HPA) online database (http://www.proteinatlas.org/) was used to discriminate between normal and GC tumor tissues^35^. Furthermore, the expression of these DEOSGs in GC was also verified in TCGA dataset as described above.

### WGCNA construction and identification of progression-annotated hub genes

A total of 279 DEOSGs in TCGA database were used to create a co-expression network using the WGCNA package in R^36,37^. Briefly, a hierarchical clustering analysis of GC tissues with numerous clinicopathological features (OS days, OS state, age, gender, grade, and stage) was applied to remove outlier samples. Subsequently, Pearson’s correlation coefficients for pairwise genes, and a weighted adjacency matrix was erected by the power function a_mn_ =|c_mn_|β (c_mn_ = Pearson’s correlation between gene m and gene n; amn = adjacency between genes m and n). After that, a suitable soft thresholding parameter β was screened to emphasize strong correlations and penalize weak correlations between genes. Then, the adjacencies were converted to a topological overlap matrix (TOM). In light of the TOM-based dissimilarity measure, the average linkage hierarchical clustering was conducted with a minimum module size of 50 for the DEOSG dendrogram, and the dissimilarity of module eigengenes was also calculated. Furthermore, two parameters [module eigengenes (MEs) and gene significance (GS)] revealed modules that were most relevant to GC progression. Hub genes comprising highly interconnected nodes within the module are regarded as functionally significant^38^. Thus, in this study, after choosing a significant module, genes with high module membership (MM > 0.8) and GS (> 0.2) were defined as candidate DEOSGs. Then, the transcriptional expression levels in normal tissues and GC samples were compared; those with significantly different expression levels were defined as the ultimate hub genes. To verify that hub genes were significantly associated with GC clinical traits, the relevance between hub genes and tumor grade was analyzed. The HPA database was used to verify the expression of the real hub genes. Meanwhile, GO and KEGG analyses using the R package were also used to identify the function and signaling pathways enriched with these hub genes.

## Results

### DEOSG identification and functional enrichment analysis

**Figure 1:**
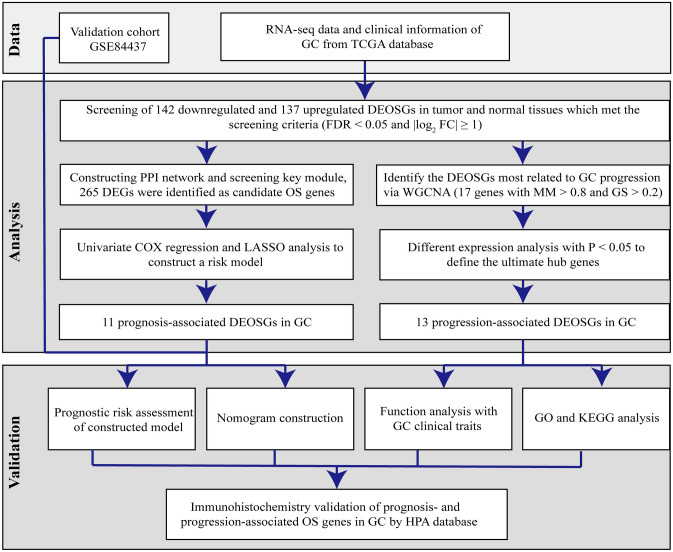
Flowchart describing the schematic overview of the study design.

**Figure 2:**
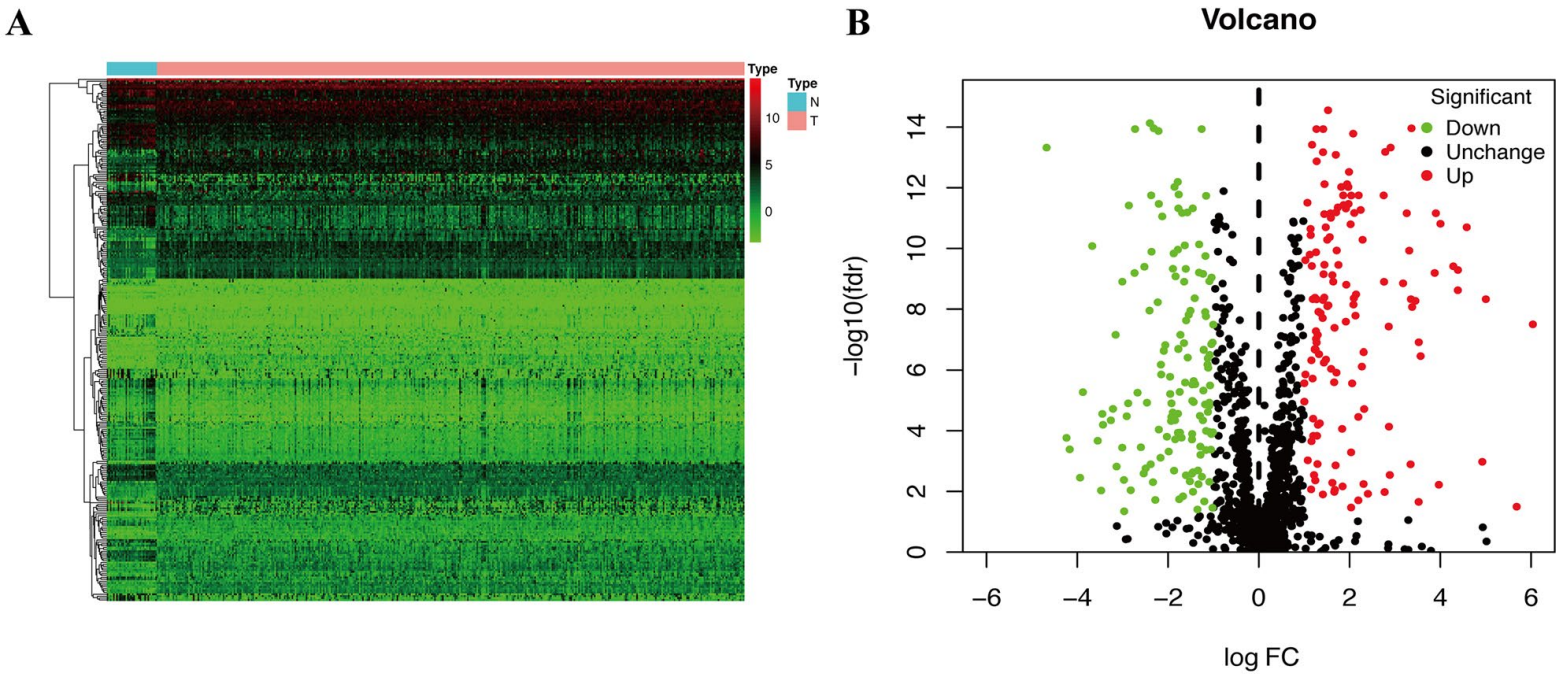
Identification of differently expressed OS genes. (**A**) Volcano plot of DEOSGs between TCGA-GC and normal stomach samples. (**B**) Heatmap of DEOSGs. Dots in green represent down-regulated genes, dots in red represent up-regulated genes, and dots in black represent unchanged genes.

**Figure 3:**
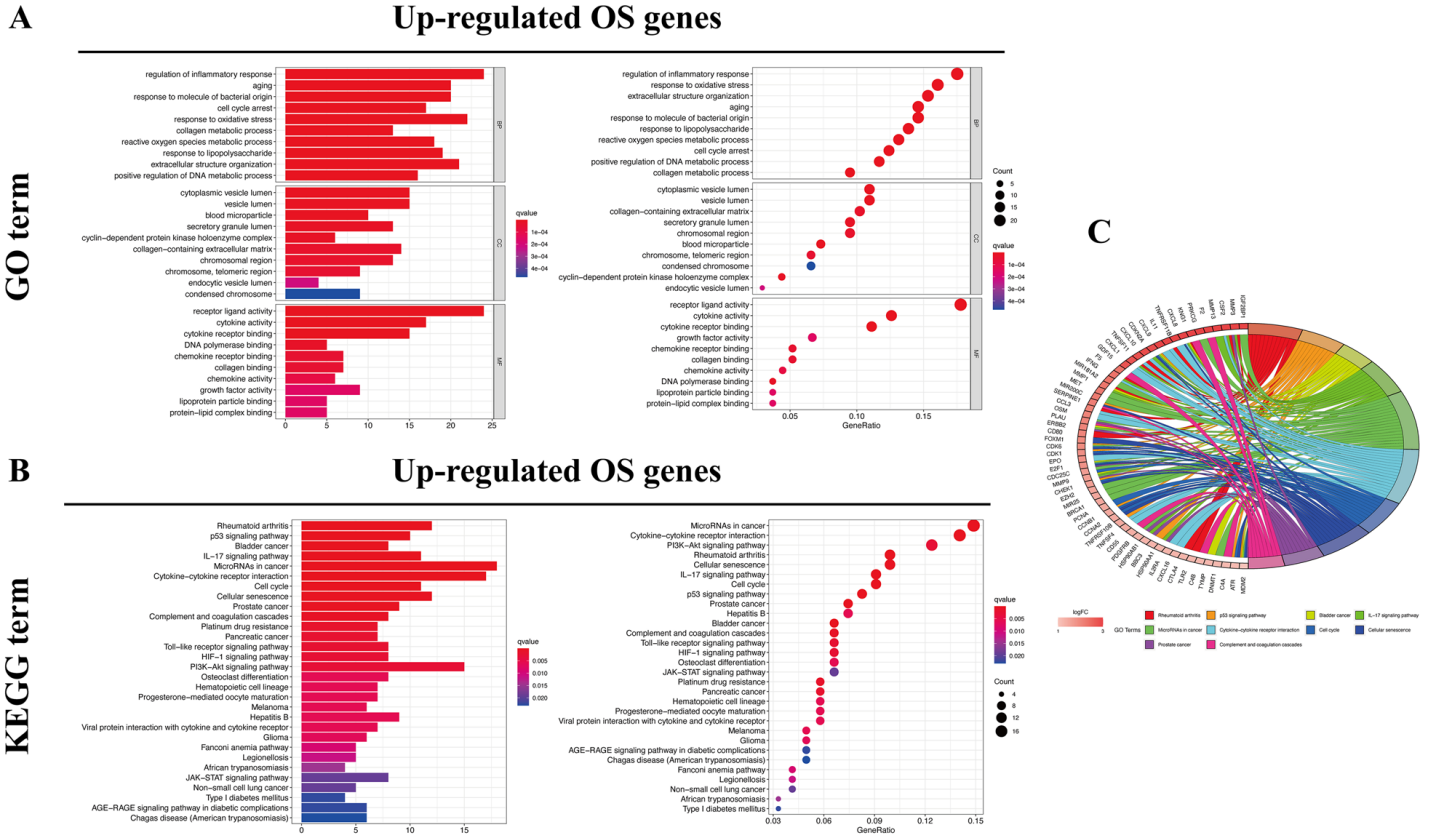
Functional enrichment analysis of up-regulated DEOSGs. (**A**) Top 10 classes of GO enrichment terms in biological process (BP), cellular component (CC), and molecular function (MF). (**B**) Top 30 classes of KEGG enrichment terms. (**C**) Circle diagram which enriched in KEGG analysis.

**Figure 4:**
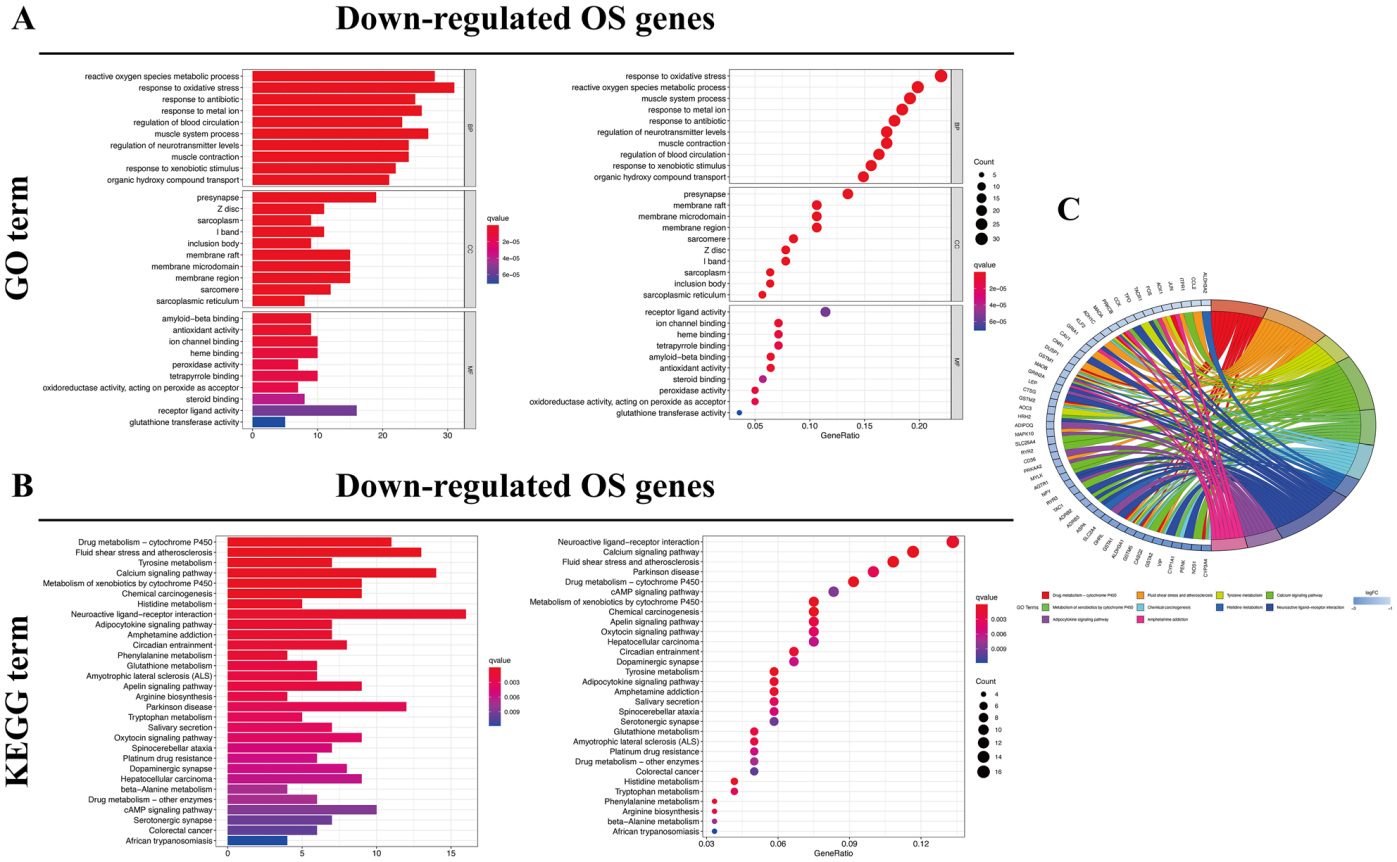
Functional enrichment analysis of down-regulated DEOSGs. (**A**) Top 10 classes of GO enrichment terms in biological process (BP), cellular component (CC), and molecular function (MF). (**B**) Top 30 classes of KEGG enrichment terms. (**C**) Circle diagram which enriched in KEGG analysis.

Bioinformatics analysis of publicly available datasets was performed according to the workflow illustrated in Fig. 1. A total of 1399 OS genes were included to compare differential expression between 32 normal stomach and 375 GC samples; 279 OS genes, comprising 142 downregulated and 137 upregulated genes, met the screening criteria (*P* < 0.05, |log2FC|> 1.0) and were identified as DEOSGs. The expression distribution of DEOSGs in GC is displayed in Fig. 2A,B. To investigate the potential functional and molecular mechanisms of these identified DEOSGs in GC, GO and KEGG analyses were performed, and the pathways these genes enriched in are displayed in Figs. 3 and 4.

### Prognosis-related DEOSG screening and construction of a genetic risk score model for patients with GC

**Figure 5:**
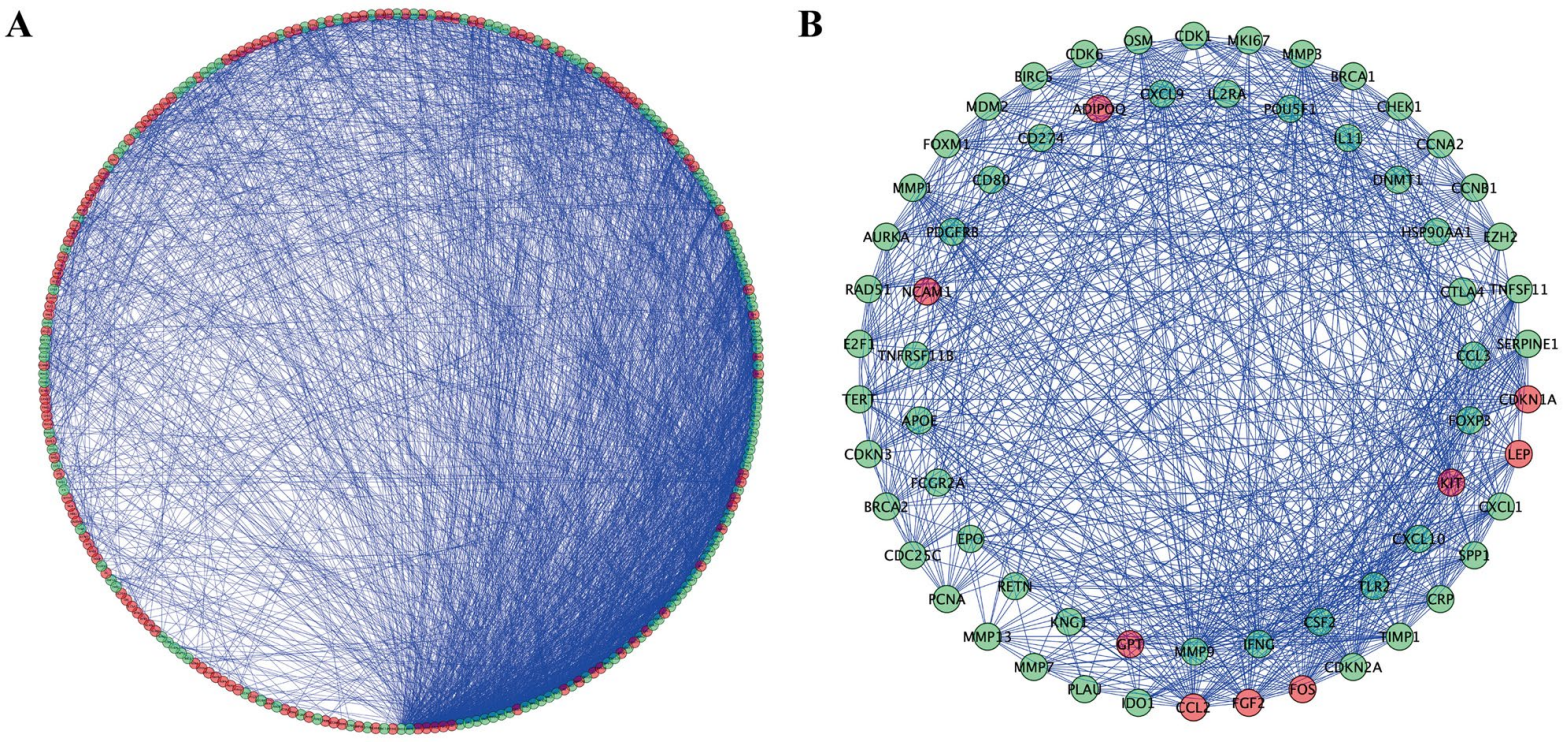
PPI network and modules screening. (**A**) PPI network of differentially expressed OS genes; (**B**) Top 2 critical modules from PPI network. Green circles represent down-regulated genes, and red circles represent up-regulated genes.

Using Cytoscape software and data from the STRING database, a PPI network with 265 nodes and 2736 edges was constructed (Fig. 5A). The MODE plugin in Cytoscape software was also used to identify the potential key modules in the network, and the top 2 significant modules were determined with 38 nodes and 329 edges, and 27 nodes and 158 edges, respectively (Fig. 5B).

**Figure 6:**
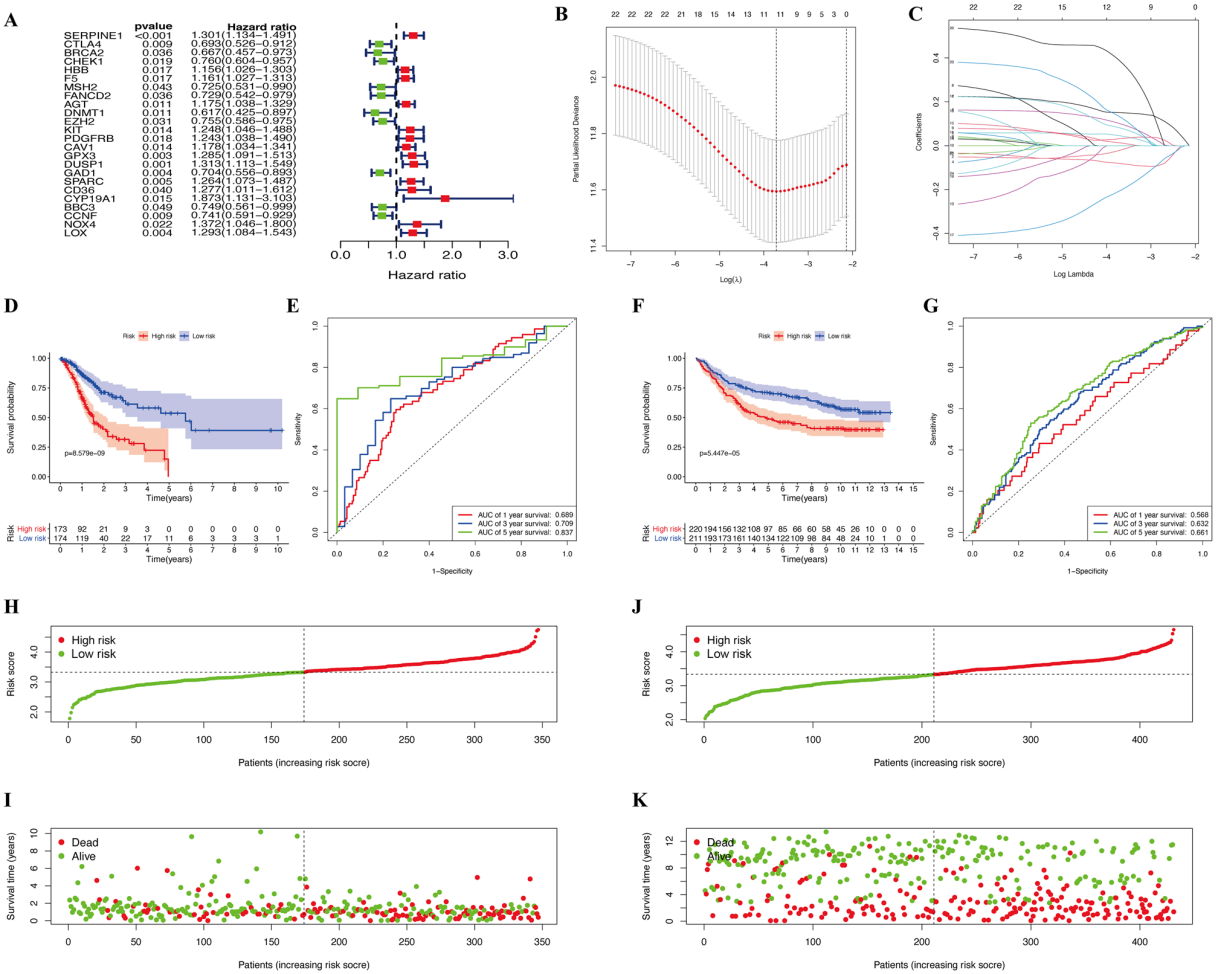
Construction of prognostic model in the TCGA and GSE84437 cohort. (**A**) Univariate Cox regression analysis for identification prognosis-associated OS genes. (**B**–**C**) LASSO analysis for determining the number of factors and constructing the prognosis prediction model. (**D**) Survival curve of TCGA cohort. (**E**) TimeROC curves for forecasting overall survival in TCGA cohort. (**F**) Survival curve of GSE cohort. (**G**) TimeROC curves for forecasting overall survival in GSE cohort. (**H**–**I**) Risk score distribution and survival status of TCGA cohort. (**J**–**K**) Risk score distribution and survival status of GSE cohort.

To further identify prognostic-related DEOSGs, 265 differentially expressed genes (DEGs) were analyzed using univariate Cox regression analysis, and 24 DEOSGs were identified as GC prognostic-associated candidate OS genes with *P* < 0.05 (Fig. 6A). Subsequently, the LASSO algorithm was employed for specific OS gene range shrinkage (Fig. 6B,C), and 11 DEOSGs [serpin family E member 1 (*SERPINE1*), cytotoxic T-lymphocyte associated protein 4 (*CTLA4*), hemoglobin subunit beta (*HBB*), coagulation factor V (*F5*), angiotensinogen (*AGT*), proto-oncogene c-KIT (*KIT*), glutathione peroxidase 3 (*GPX3*), glutamate decarboxylase 1 (*GAD1*), cytochrome P450 family 19 subfamily A member 1 (*CYP19A1*), Bcl-2-binding component 3 (*BBC3*), and NADPH oxidase 4 (*NOX4*)] were selected to calculate the risk score; all patients with GC were separated into low- and high-risk groups according to the median risk score (Fig. 6H-K). The coefficients of the 11 DEOSGs are shown in Table 1. As indicated in Fig. 6D,F, the overall survival of patients with GC was significantly decreased with an increased risk score in both TCGA and GSE84437 databases. In addition, time-dependent ROC analysis indicated that the prediction model was quite credible, with the area under the ROC curve (AUC) reaching 0.837 at 5 years in TCGA database (Fig. 6E). Similar accuracy was also validated in the GSE84437 cohort, with the AUC reaching 0.661 at 5 years (Fig. 6G), which indicated that this prognostic model had moderate specificity and sensitivity. However, while GC patients were separated into the alive and dead subgroups, our increasing risk score no longer showed a clear correlation with GC patients’ survival time (Fig. 6I,K), which may suggest that our risk model can only predict the survival rate of the total population but cannot be used to predict the specific survival time of GC patients.

**Table 1:** Eleven prognosis-associated OS genes with GC in the TCGA dataset were identified by LASSO analysis.

OS name	Univariate Cox regression analysis				LASSO coefficient
	HR	Lower 95% CI	Upper 95% CI	*P* value	
SERPINE1	1.3005	1.1340	1.4915	0.0002	0.1520
CTLA4	0.6929	0.5263	0.9121	0.0089	−0.0772
HBB	1.1564	1.0263	1.3029	0.0170	0.0506
F5	1.1611	1.0270	1.3126	0.0170	0.1119
AGT	1.1746	1.0383	1.3288	0.0106	0.0176
KIT	1.2478	1.0463	1.4880	0.0138	0.0907
GPX3	1.2850	1.0913	1.5131	0.0026	0.0283
GAD1	0.7044	0.5556	0.8931	0.0038	−0.2259
CYP19A1	1.8730	1.1306	3.1029	0.0148	0.4194
BBC3	0.7485	0.5610	0.9988	0.0491	−0.0313
NOX4	1.3723	1.0461	1.8001	0.0223	0.1537

**Figure 7:**
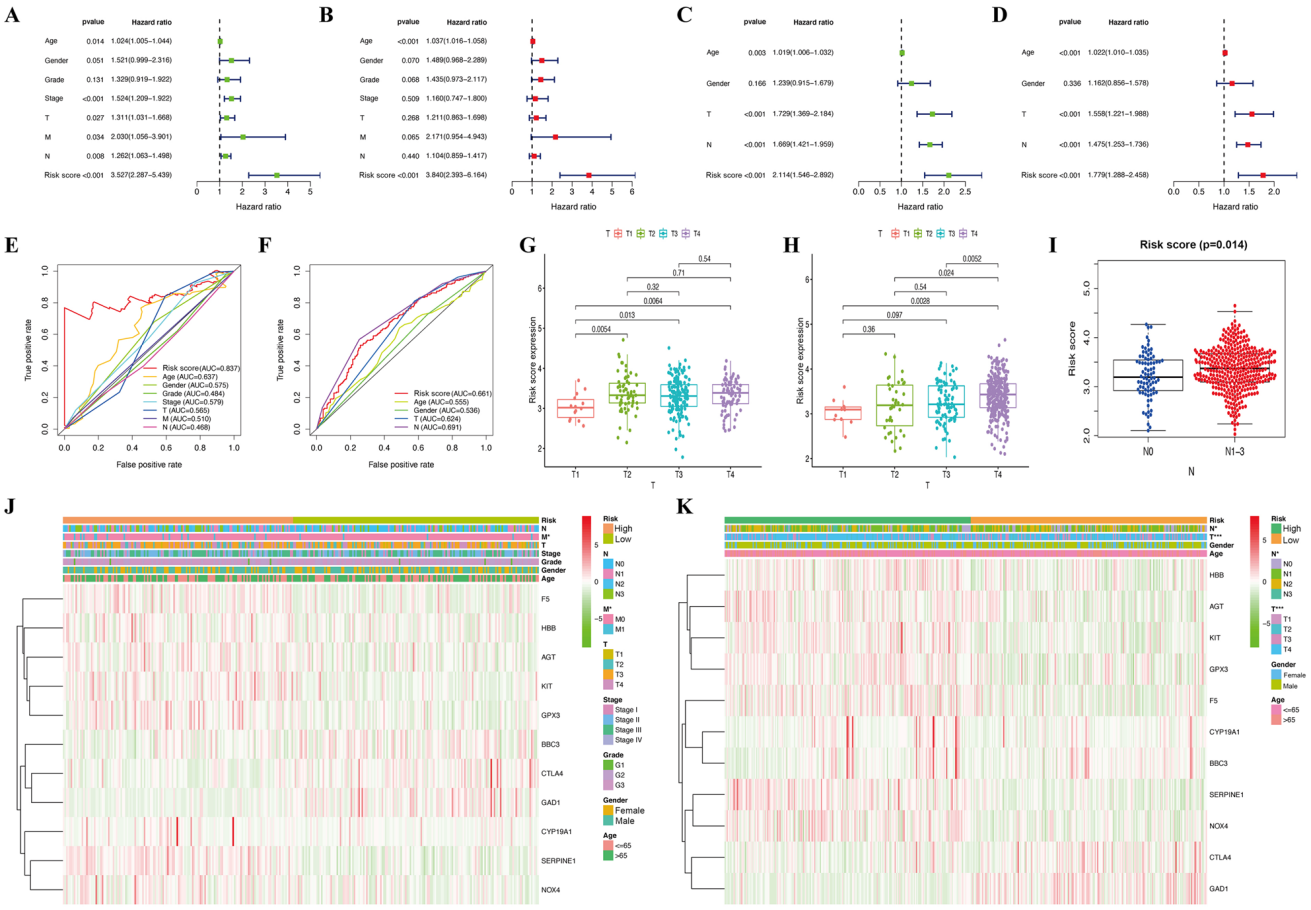
Efficacy evaluation of constructed prognostic model. Univariate (**A**) and multivariate (**B**) Cox regression analysis of the clinicopathological features in TCGA cohort. Univariate (**C**) and multivariate (**D**) Cox regression analysis of the clinicopathological features in GSE84437 cohort. ClinicalROC curves for forecasting overall survival in TCGA (**E**) and GSE84437 (**F**) cohort. (**G**) The relationship between the risk scores and T stage in TCGA cohort. The relationship between the risk scores and T stage (**H**) or N stage (**I**) in GSE84437 cohort. The heatmap shows the distribution of clinicopathological features and OS genes expression in TCGA (J) and GSE84437 (**K**) cohort. Columns in green represent down-regulated genes, columns in red represent up-regulated genes, and columns in white represent unchanged genes.

To determine whether the risk signature was an independent prognostic factor, univariate and multivariate Cox regression analyses were also performed. The risk score was an independent prognostic feature that was significantly connected with GC prognosis in both TCGA and GSE84437 databases (Fig. 7A-D). The ROC curve over 5 years showed that the prognostic model had a better predictive accuracy than other clinical features in the TCGA cohort (Fig. 7E). In the GSE84437 cohort, the prognostic model also showed better forecast performance than age, gender, and T stage (Fig. 7F). Moreover, the correlation between risk score and each clinicopathological characteristic was evaluated; patients with GC in the T1 stage were significantly related to a lower risk score in the TCGA cohort (Fig. 7G). Meanwhile, the risk score was also associated with the different T and N stages of patients with GC in the GSE84437 cohort, and GC patients in the T4 or N1-3 stage were significantly related to a higher risk score (Fig. 7H-I). The heatmap revealed the expression of the 11 specific DEOSGs in each subgroup, and there were significant differences between the two risk subgroups with respect to the M stage in TCGA database (Fig. 7J). In the GSE84437 cohort, the expression trends of DEOSGs were quite similar to those in TCGA cohort, and there were significant differences between the two risk subgroups with respect to T and N stage (Fig. 7K).

**Figure 8:**
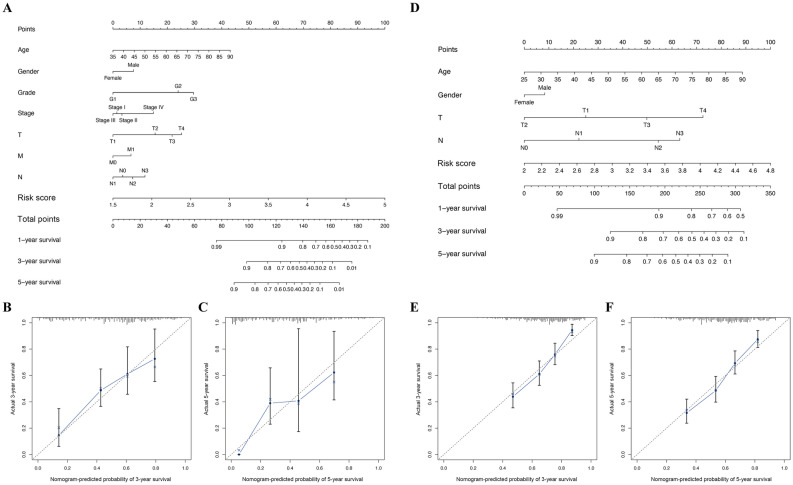
Nomogram plots construction. Nomogram of risk score and other clinical factors for predicting GC 1-, 3-, and 5-year overall survival in TCGA (**A**) and GSE84437 (**D**) cohort. (**B**–**C**) The calibration plot of the nomogram in TCGA cohort. (**E**–**F**) The calibration plot of the nomogram in GSE84437 cohort.

A nomogram plot is another quantitative model to predict clinical outcomes of patients with GC. Thus, a nomogram plot was developed based on the risk score and other clinical characteristics, which allowed the calculation of the survival probabilities of each patient with GC at 1, 3, and 5 years (Fig. 8A,D). The calibration plots indicated good conformity between predicted and observed outcomes at 3 and 5 years in both TCGA and GSE84437 cohorts (Fig. 8B,C,E,F). These results indicated that the prognostic model showed great promise for predicting GC outcomes and clinical features.

### Evaluation of the expression level of prognosis related DEOSGs in patients with GC

To further explore the transcriptional pattern of DEOSGs in patients with GC, the expression value of each key gene was extracted from TCGA database and a violin plot and heatmap was constructed. As shown in Supplement file 2A,B, the results indicated that *SERPINE1, CTLA4, F5, AGT, GAD1, CYP19A1, BBC3*, and *NOX4* were significantly overexpressed in GC samples, while the expression patterns of *HBB, KIT*, and *GPX3* were decreased compared to those in normal tissues. Similar results were obtained by analyzing the protein expression levels of the key DEOSGs in accordance with the immunohistochemistry results from the HPA database (Supplement file 2C).

### Identification of hub DEOSGs for tumor grade by constructing a weighted co‑expression network

**Figure 9:**
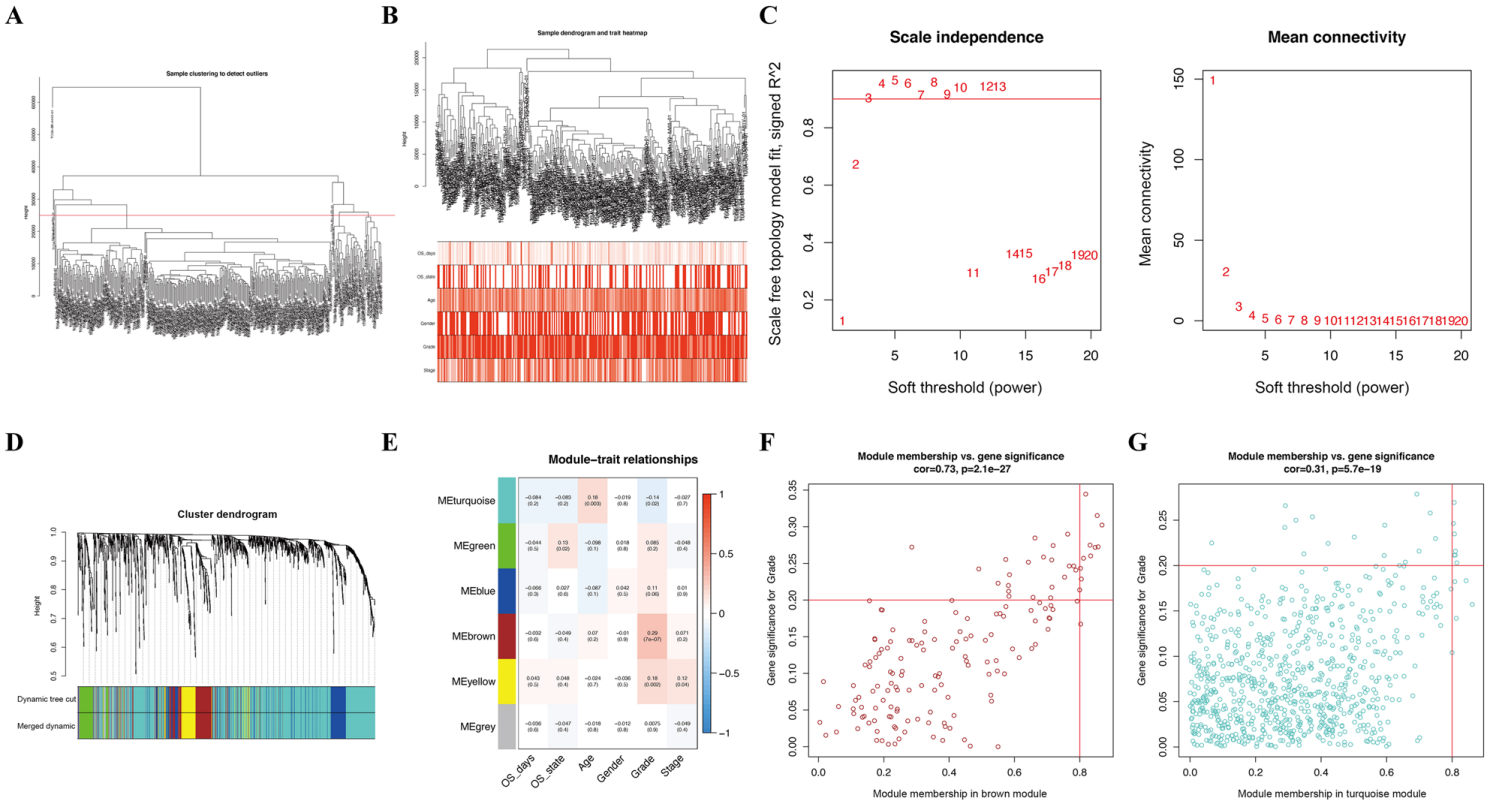
WGCNA analysis. (**A**) Samples clustering of OS genes from TCGA database to detect outliers. (B) Clustering dendrogram of GC samples and associated clinical traits. (**C**) The scale-free fit index for soft-thresholding powers. (**D**) A dendrogram of the differentially expressed genes clustered based on different metrics. (**E**) A heatmap showing the correlation between the gene module and clinical traits. Scatter plot of module eigengenes in brown (**F**) and turquoise (**G**) modules.

**Figure 10:**
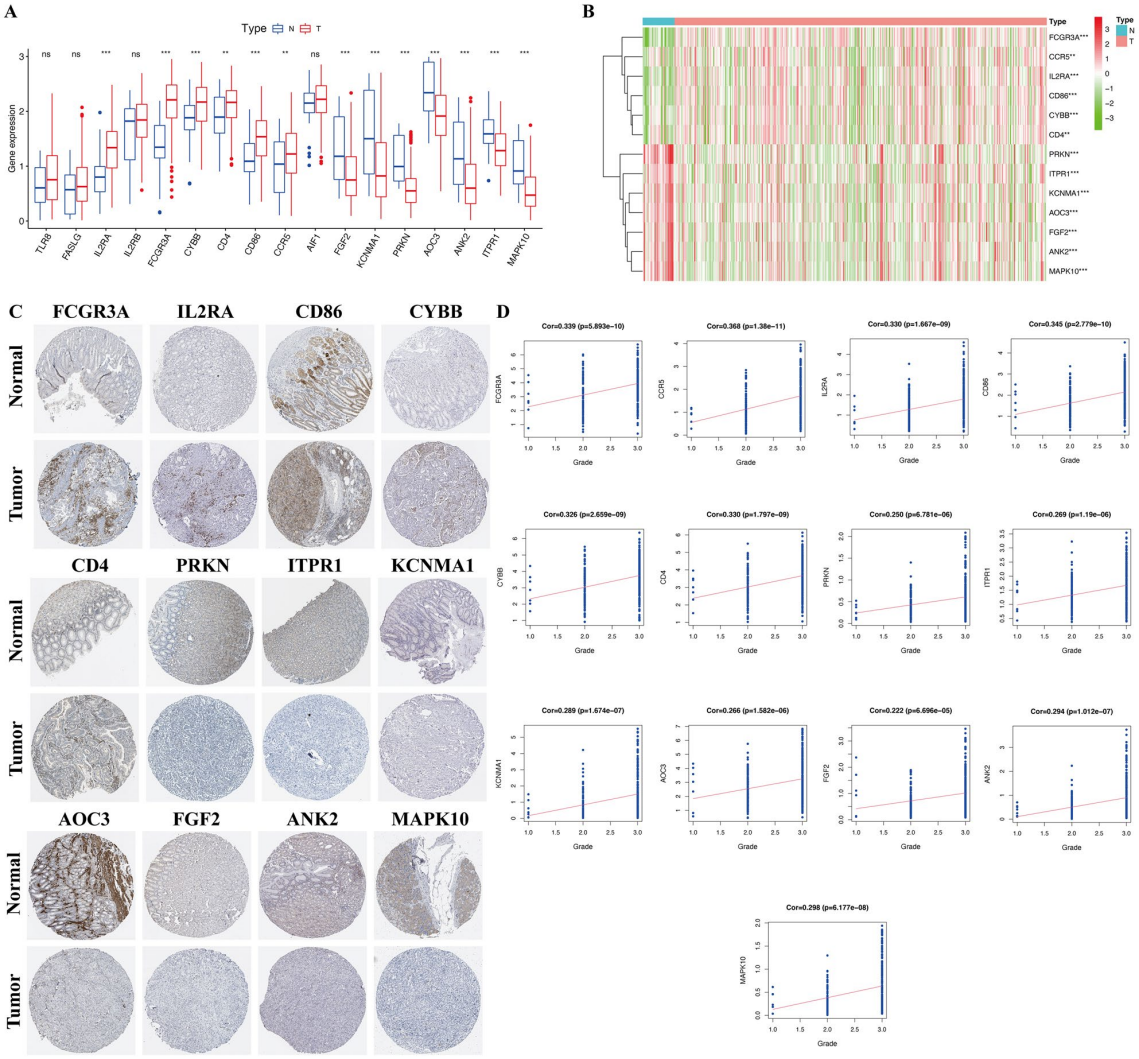
Connections between the expression of progression-associated OS genes and GC grade. (**A**) The mRNA expression pattern of progression-associated OS genes in TCGA cohort. (**B**) The heatmap reveals the transcription expression of progression-associated OS genes in TCGA cohort. (**C**) HPA database verifies the protein expression of hub progression-associated OS genes. (**D**) Correlation analysis between 13 progression-associated OS genes expression and tumor grade.

Furthermore, WGCNA of 401 GC samples with complete clinical data from TCGA database was performed on 279 DEOSGs. Patients with GC with six types of clinical characteristics, including overall status, overall survival time, age, sex, tumor grade, and TNM stage were included for analysis, and probes with variances ranked in the top 25,000 were subjected to modules (Fig. 9A,B). To construct a scale-free network, the power of β = 3 (scale free R^2^ = 0.90) was selected as the soft threshold (Fig. 9C); a total of six co‑expressed modules were identified (Fig. 9D). Subsequently, to identify the module that was most related to GC progression, each module was assigned a different color. Among the modules, the brown module was specifically positively connected with tumor grade (*P* < 0.05), and genes in the turquoise module were negatively related to GC progression (*P* < 0.05, Fig. 9E). Thus, the data from these two modules were identified as candidate genes of interest in the training set. To further screen the most significant hub genes that were relevant to the tumor progression of GC, 17 genes with remarkable connectivity (MM > 0.8 and GS > 0.2) were identified in the brown and turquoise modules (Fig. 9F,G). Subsequently, the transcription of 17 candidate genes between GC samples and normal tissues were compared (Fig. 10A). Ultimately, 13 significant DEOSGs were identified as “real" GC progression-associated hub genes for further validation.

### Validation of the relationship between progression annotated DEOSGs and clinicopathological features of GC

**Figure 11:**
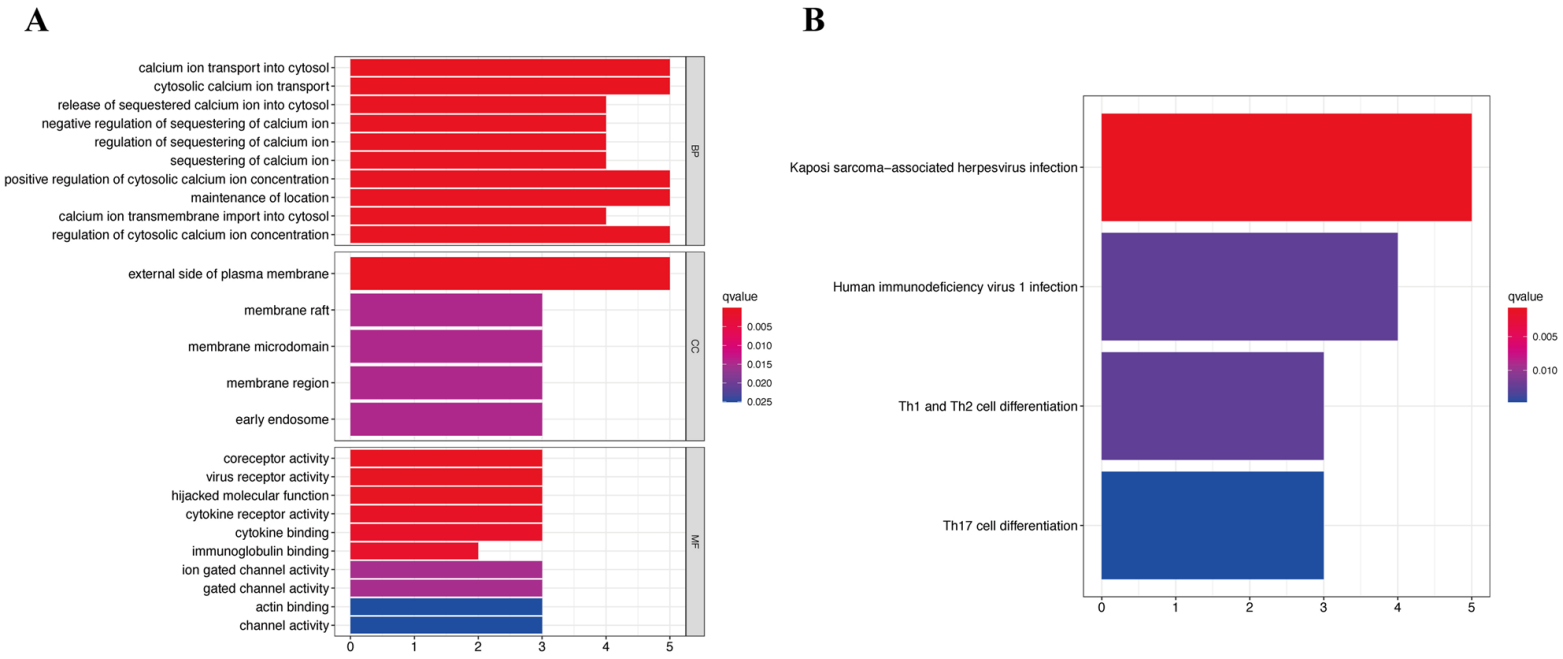
Function analysis of progression-associated OS genes. (**A**) Top 10 classes of GO enrichment terms about 13 hub genes in biological process (BP), cellular component (CC), and molecular function (MF). (**B**) KEGG enrichment terms about 13 hub progression-associated OS genes.

Based on TCGA database, the expression of the genes Fc fragment of IgG receptor IIIa (*FCGR3A*), C–C motif chemokine receptor 5 (*CCR5*), interleukin-2 receptor alpha (*IL2RA*), cluster of differentiation 86 (*CD86*), cytochrome B-245 beta chain (*CYBB*), and cluster of differentiation 4 (*CD4*) were significantly elevated, while the expression of parkin RBR E3 ubiquitin protein ligase (*PRKN*), inositol 1,4,5-trisphosphate receptor type 1 (*ITPR1*), potassium calcium-activated channel subfamily M alpha 1 (*KCNMA1*), amine oxidase copper containing 3 (*AOC3*), fibroblast growth factor 2 (*FGF2*), ankyrin-2 (*ANK2*), and mitogen-activated protein kinase 10 (*MAPK10*) was significantly decreased in GC tissues (Fig. 10B). Immunohistochemistry images obtained from the HPA database also demonstrated the same expression trend of the 13 hub genes (Fig. 10C). Kaplan–Meier analysis was implemented to determine the capacity of hub genes to predict GC prognosis; only *MAPK10* was significantly correlated with GC overall survival (Supplement file 3). However, the regression analysis indicated that all 13 hub genes were strongly associated with GC grade (Fig. 10D), which indicated that these DEOSGs were mainly involved in GC progression, but with poor prognostic ability. Similar results were also confirmed in the relationship between the expression of the 13 hub DEOSGs and clinicopathological characteristics of GC. As shown in Table 2, the 13 hub genes were all significantly connected with GC grade (Supplement file 4A), while all 12 DEOSGs, except *PRKN*, were significantly related to the T stage of patients with GC (Supplement file 4C). Furthermore, the expression of *FCGR3A, CCR5, FGF2, KCNMA1, AOC3, ANK2,* and *MAPK10* was also significantly associated with patients’ TNM stage; only the expression of *KCNMA1* was associated with M stage (Supplement file 4B,D). Furthermore, GO and KEGG analyses were also used to identify the potential mechanisms of the 13 real hub genes in GC progression. GO enrichment results indicated that these genes were mainly enriched in calcium ion transport into the cytosol, external side of the plasma membrane, and coreceptor activity (Fig. 11A). Additionally, KEGG pathway analysis indicated that the 13 hub genes were mostly enriched in Kaposi sarcoma-associated herpesvirus infection, human immunodeficiency virus 1 infection, and Th1, Th2, and Th17 cell differentiation (Fig. 11B).

**Table 2:** Correction between 13 hub progression-associated OS genes expression and clinicopathological characteristics of GC.

Clinical variables	P-value												
	IL2RA	FCGR3A	CYBB	CD4	CD86	CCR5	FGF2	KCNMA1	PRKN	AOC3	ANK2	ITPR1	MAPK10
Age	0.341	0.095	0.657	0.412	0.690	0.547	0.048	0.023	0.008	0.015	0.008	0.015	0.026
Gender	0.811	0.390	0.393	0.588	0.312	0.158	0.586	0.546	0.607	0.630	0.464	0.931	0.377
Tumor grade	2.115e − 08	1.144e − 08	2.426e − 08	6.316e − 09	2.159e − 09	1.495e − 10	6.117e − 04	1.139e − 08	3.142e − 04	1.339e − 05	7.204e − 06	2.237e − 05	5.315e − 06
TNM stage	0.143	0.030	0.145	0.059	0.056	0.015	0.028	0.004	0.437	0.001	0.004	0.087	0.034
T stage	0.039	1.999e − 04	0.005	0.010	0.002	0.003	7.24e − 04	3.55e − 04	0.529	2.205e − 04	8.653e − 05	0.027	0.019
M stage	0.411	0.586	0.818	0.620	0.201	0.351	0.500	0.020	0.105	0.456	0.316	0.873	0.770
N stage	0.154	0.277	0.258	0.258	0.181	0.326	0.367	0.946	0.267	0.982	0.406	0.101	0.311

## Discussion

As a worldwide malignant tumor, GC is reported to have heterogeneous characteristics in the digestive system^39^. Although many novel diagnostic techniques and surgical skills have been developed in recent years, they are not always sufficient. Therefore, it is imperative to identify GC prognosis-related molecules and determine the mechanism of tumor progression. In the present study, 279 DEOSGs were identified based on TCGA database, and relevant biological pathways and PPI networks were systematically identified for these genes. Pathway enrichment analysis revealed that the DEGs were significantly correlated to the progression of several types of tumors, such as melanoma, glioma, and bladder, prostate, and pancreatic cancers. Furthermore, these DEOSGs were significantly enriched in several biological processes, including inflammatory response, reactive oxygen species metabolic process, and response to oxidative stress. All of these biological processes have been reported to be significantly correlated with tumorigenesis and progression^40–42^. Thus, these DEGs were significantly associated with GC progression and critical for the comprehensive evaluation of the mechanism of these DEOSGs.

In addition, the prognosis related DEOSGs were screened by univariate Cox and LASSO regression analysis, and a total of 11 DEOSGs: *SERPINE1, CTLA4, HBB, F5, AGT, KIT, GPX3, GAD1, CYP19A1, BBC3,* and *NOX4* were identified with a good prognosis in patients with GC. The expression patterns of these 11 DEOSGs on mRNA and protein levels using TCGA expression data and HPA database revealed that *SERPINE1, CTLA4, F5, AGT, GAD1, CYP19A1, BBC3,* and *NOX4* were overexpressed, while *HBB, KIT,* and *GPX3* were downregulated in GC tissues. These findings are mostly consistent those of previous studies showing that the expression of *SERPINE1* and *CTLA4* is elevated in gastric adenocarcinoma, and as a tumor carcinogenic gene, the overexpression of *SERPINE1* is significantly associated with GC aggressiveness and inferior overall survival^43,44^. In addition, *GAD1* is overexpressed in lung adenocarcinoma and plays a virtual role in tumor progression^45^; however, its role in GC outcome is unclear.

To further identify whether these specific DEOSGs could be used as prognostic factors, a novel prognostic prediction model was constructed based on these 11 hub genes. To our knowledge, this is the first OS-associated risk model for prognostication. Univariate and multivariate Cox regression analyses showed that the risk model was an independent prognostic factor with a robust prognostic value for GC. In addition, the survival analyses and ROC analyses also confirmed the major advantage of its biological implications for predicting GC prognosis. A similar scenario was also observed in the nomogram analysis that risk signature played a virtual role in predicting the overall survival of patients with GC, and its inspection efficiency was much better than that of other clinicopathological features. These explorations, for the first time, demonstrate the prognostic value of an OS gene-dependent risk model for patients with GC and provide a novel direction for further research.

To a great extent, OS plays a critical role in various stages of carcinogenesis and cancer progression^46,47^. In recent years, many GC studies have identified that OS may be closely associated with progression owing to resulting DNA damage^48^. However, in our previous study, the risk model, which was developed using the hub prognosis associated DEOSGs, was only associated with TNM stage of patients with GC, but there was no significant correlation with tumor grade. Therefore, in follow-up studies, we defined DEOSGs that were closely related to tumor progression. WGCNA is a widely used approach to identify potential biomarkers of interest^49,50^. In the present study, 13 real hub genes (*FCGR3A, CCR5, IL2RA, CD86, CYBB, CD4, PRKN, ITPR1, KCNMA1, AOC3, FGF2, ANK2,* and *MAPK10*) tightly associated with GC progression were identified, and a series of bioinformatic analyses showed that these genes were both highly correlated with GC grade and may be potential biomarkers for predicting tumor stage. Moreover, considering the critical role of tumor progression in the overall survival of patients with GC, the prognostic value of these hub genes was assessed; *MAPK10* was significantly correlated with patients’ overall survival. Compared to previous studies, Gu’s and Ying’s group, who focused on the prognostic value of *MAPK10*, also confirm our conclusion that *MAPK10* is frequently downregulated in GC cell lines^51^ and predicts tumor progression and prognosis^52^. The functional and pathway enrichment analysis showed that these genes were mainly enriched in terms that were related to malignancy progression, including calcium ion transport into cytosol^53^, coreceptor activity^54^, and Kaposi sarcoma-associated herpesvirus infection^55^, which may provide a perspective for exploring the role of prognosis-related DEOSGs in GC.

Nonetheless, this study has limitations. First, this study was designed as a retrospective analysis; therefore, more prospective studies should be performed to verify these results. Second, the results lack in vitro or in vivo exploration to confirm the reliability of the mechanistic analysis. Therefore, in the future, a number of experiments will be conducted to demonstrate the mechanistic connections between these genes and GC progression.

In conclusion, after a series of bioinformatic analyses and verifications, 11 prognosis-associated DEOSGs and 13 progression-associated DEOSGs were identified, which were related to the overall survival or tumor grade of patients with GC. A prognostic model was also constructed with powerful predictive effects. As far as we know, this is the first report of the construction of an OS-associated prognostic model for malignancies. This study provides novel research targets for studying the pathogenesis and progression of patients with GC.

## Supplementary Information

Supplementary Information 1.

Supplementary Information 2.

Supplementary Information 3.

Supplementary Information 4.

## Data Availability

The data used to support the findings of this study are available from the corresponding author upon request.
