# Supplementary material for: Integrated analysis identifies oxidative stress genes associated with progression and prognosis in gastric cancer
Source: Sci Rep. 2021 Feb 8;11:3292. doi: 10.1038/s41598-021-82976-w (PMC7870842; doi:10.1038/s41598-021-82976-w)

**Integrated analysis identifies oxidative stress genes associated with progression and prognosis in gastric cancer**

Running title: Value of OS genes in gastric cancer

Zhengyuan Wu^1^, Lin Wang^2^, Zhenpei Wen ^2^, Jun Yao^2, 3*^

1. Department of Orthopedics Trauma and Hand Surgery, The First Affiliated Hospital of Guangxi Medical University, Nanning, 530021, China
2. Department of Bone and Joint Surgery, The First Affiliated Hospital of Guangxi Medical University, Nanning, 530021, China
3. Guangxi Collaborative Innovation Center for Biomedicine, Guangxi Medical University, Nanning, 530021, China

Zhengyuan Wu and Lin Wang contributed equally to this manuscript.

* Co-Corresponding authors.

Jun Yao: Tel: +86-13907867699. Fax: +86-07715350189. Email address: yaojun800524@163.com

**Supplement File 4** shows the relationship between 13 hub progression-associated OS genes expression and clinicopathological characteristics of GC in TCGA cohort.


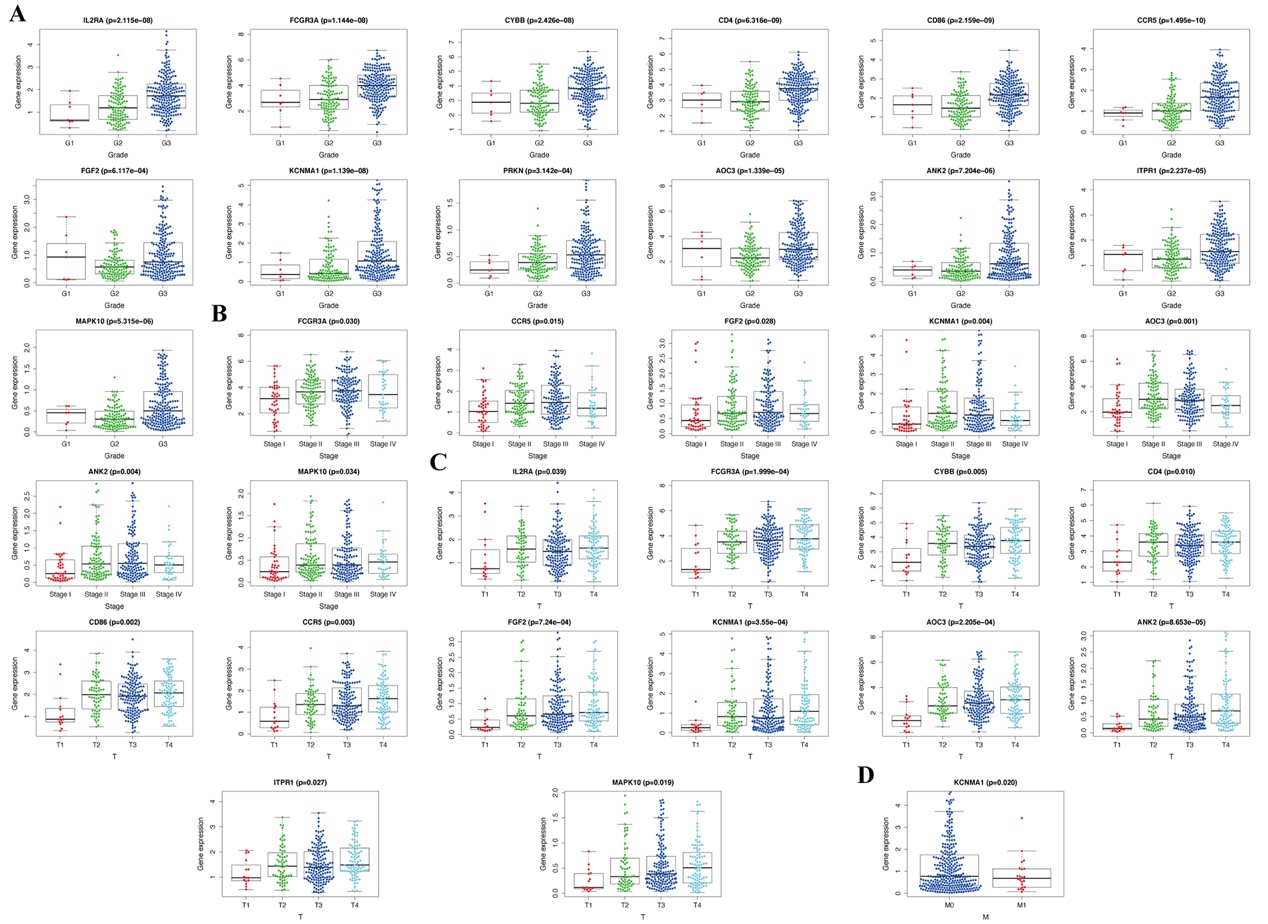

Supplement: Supplementary file 4 — Supplementary Information 4. [file 41598_2021_82976_MOESM4_ESM.docx]
